# Dental-gingival remodeling with BOPT no-prep veneers

**DOI:** 10.4317/jced.54463

**Published:** 2017-12-01

**Authors:** Rubén Agustín-Panadero, Daniel Ausina- Escrihuela, Lucía Fernández-Estevan, Juan-Luis Román-Rodríguez, Joan Faus-López, María-Fernanda Solá-Ruíz

**Affiliations:** 1 DMD, PhD, Associate Professor, Department of Dental Medicine, Faculty of Medicine and Dentistry, University of Valencia, Spain; 2 DMD, In private dental practice, Spain; 3 DMD, PhD, Director of Valencia Dental Research Institute (IVIO), Valencia, Spain; 4 DMD, PhD, MD, Adjunct Professor, Department of Dental Medicine, Faculty of Medicine and Dentistry, University of Valencia, Spain

## Abstract

Recent years have seen increasing demand for treatments aimed at improving dental esthetics. In this context, both patients and dentists prefer to preserve dental structures as far as possible; thanks to technological advances, especially in adhesive dentistry, new materials and minimally invasive techniques such as “no-prep” (no preparation) veneers have made this possible. Nevertheless, no-prep veneers have specific indications and suffer certain disadvantages. Objectives: This clinical case describes the rehabilitation of the upper anterior region by means of no-prep veneers, with BOPT (Biologically Oriented Preparation Technique) cervical margins. The patient had requested an aesthetic treatment to improve irregularities of the gingival margins associated with the presence of diastemata resulting from microdontia.

** Key words:**BOPT, micro-veneers, hybrid ceramic, ultra-fine veneers, diastemata, without prosthetic finish line, no-prep.

## Introduction

The last 30 years (1) has seen the introduction of ceramic veneers aimed at improving the shape, alignment, and color of teeth as a response for patients increasing demands for improved dental esthetics. To begin with, these were designed for use without any kind of preparation, but later, in response to the deficiencies of materials and techniques, teeth underwent a milling procedure to reduce their thickness and so accommodate the ceramic veneers, ensuring esthetic outcomes and the treatment’s long-term durability (1). However, patients and clinicians’ concern to preserve a maximum quantity of healthy dental structures has led to further research into new materials and techniques aimed at maximizing dental conservation. New materials have appeared, especially in adhesive dentistry, that have made it possible to fabricate veneers that do not require any tooth preparation, while providing excellent long term function and esthetics (2).

Objectives: This clinical case report describes the placement of six hybrid ceramic veneers without preparation of the dental structure, applying BOPT (Biologically Oriented Preparation Technique) for purposes of gingival tissue modeling and closure of diastemata caused by microdontia.

## Case Report

**Figure 1:**
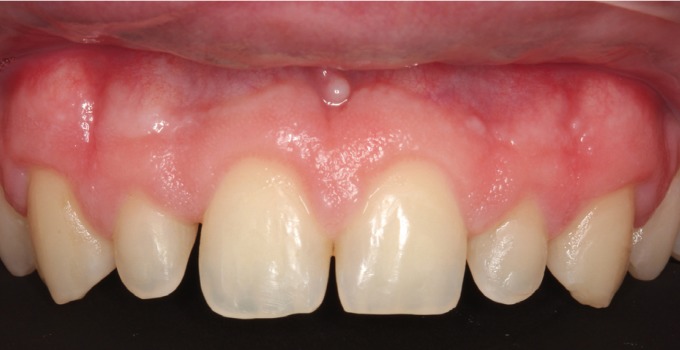
Patient’s appearance on first visit to the clinic.

**Figure 2:**
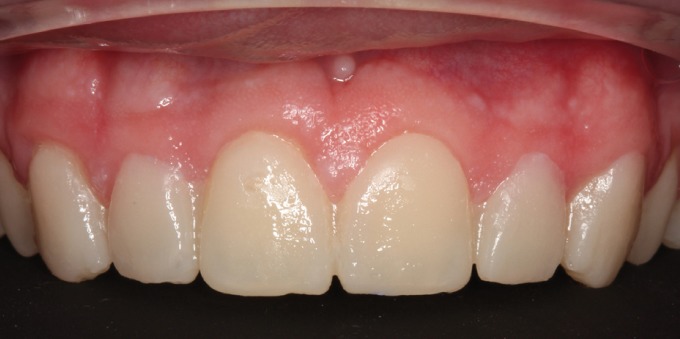
Mock-up in position in mouth.

A male patient aged 25 years sought treatment to improve his dental esthetics because he was concerned about small tooth sizes in the upper anterior region (Fig. 1). A pre-treatment mock-up was fabricated on the basis of models and a diagnostic wax-up (2,3) (Fig. 2). The treatment plan consisted of applying no-prep veneers without tooth preparation with BOPT-type morphology in the cervical area in order to regularize the position of the gingival margins (3,4) and close diastemata.

As no kind of dental or gingival preparation was performed, impressions were taken with addition silicon (Elite HD+ Putty Soft Normal Set, Zhermack®, Rovigo, Italy) using double-mix impression technique (Wash Technique) with double-cord retraction (Ultrapak®, Ultradent, Utah, United States), one cord of 0.89mm diameter at the base of the groove and the other of 1.6mm diameter to the crown. The latter was removed at the moment when the liquid impression material was injected (Elite HD+ Light Body Normal Set, Zhermack®) (4).

**Figure 3:**
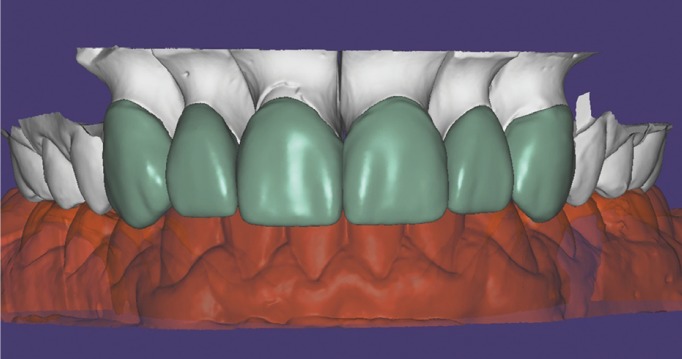
Digital design of Vita Enamic HT veneers.

**Figure 4:**
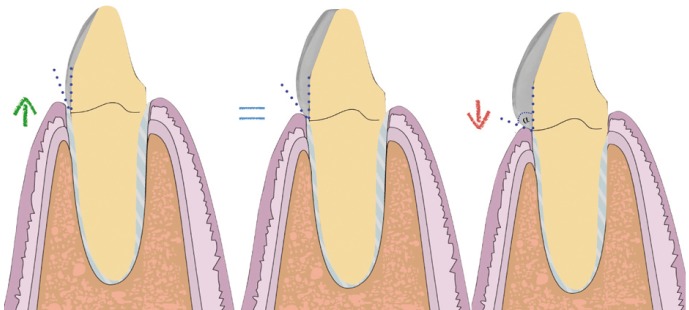
Modification of the gingiva with respect to the prosthetic emergency.

The impression was cast in type IV plaster (Elite rock, Zhermack®) to obtain a physical master model. Intermaxillary registers and cranio-maxillary transfers were taken and mounted on an ARL Dentatus semi-adjustable articulator set-up (Dentatus USA Ltd, New York, United States). The physical master model was then digitalized using an extraoral scanner and dedicated software (3Shape CAD Design, Copenhagen, Denmark) and stored as an STL file. This was used to design a virtual wax-up on the digital model for fabricating veneers for teeth 13, 12, 11, 21, 22 and 23 (Fig. 3). As the teeth and gingival tissue underwent no preparation, it was decided to make micro-veneers (mean thickness 0.2mm) using a hybrid ceramic, VITA Enamic HT (VITA Zahnfa-brik, Bad Säckingen, Germany) with BOPT-type prosthetic cervical emergence. In order to obtain the correct gingival scallop shape, veneers were given different cervical emergence morphologies; in this way, veneers for teeth 13, 11 and 21 presented a flattened cervical emergence with an angle of ≤45º between the dental axis and the prosthetic piece in order to avoid modifying the gingival position for these teeth. For teeth 12, 22 and 23, where the gingival margin position was more coronal, it was decided to create a prosthetic emergence profile >60°, in order to bring about controlled ischemia in the gingival area, and so displace the level of the gingival margin slightly towards apical. All veneers were fitted to the cervical level at the cementoenamel junction, as the main objective of these restorations was not to invade the biological space in vertical direction but to manage the convexity of the tooth’s anatomical crown so that the gingiva would adapt to the crown shape in either apical or coronal direction (Fig. 4).

**Figure 5:**
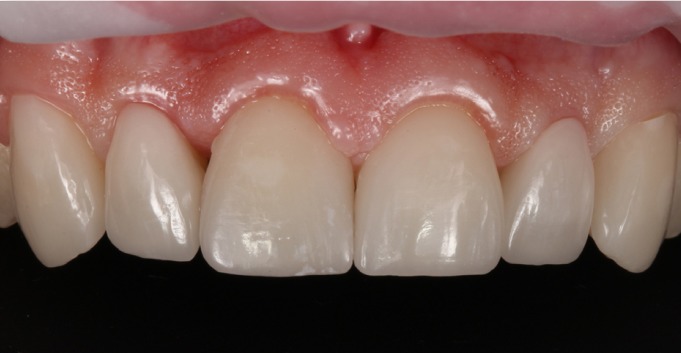
Checking the veneers in the mouth. N.B. ischemia at tooth 12 in response to pressure from BOPT emergence in order to raise the gingival margin.

**Figure 6:**
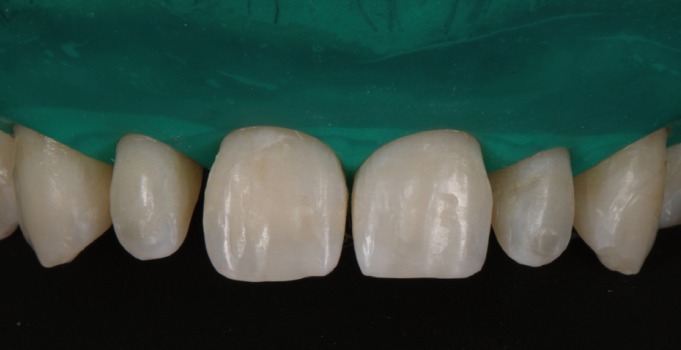
Isolation with rubber dam.

**Figure 7:**
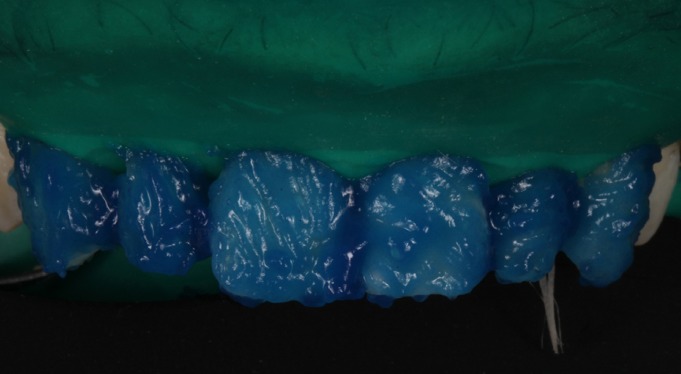
Etching the teeth with orthophosphoric acid.

**Figure 8:**
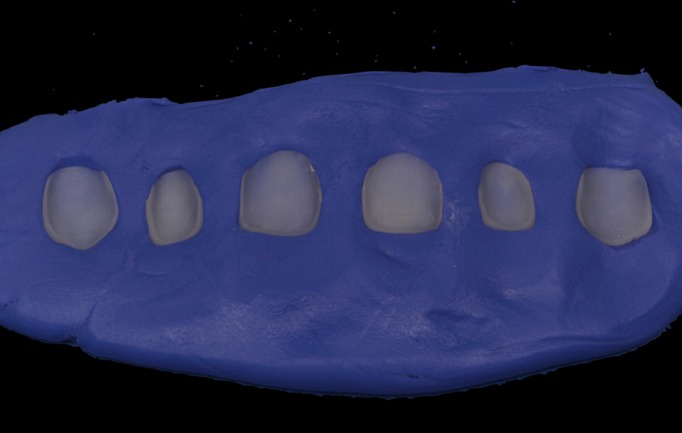
Veneers positioned in putty before etching with hydrofluoric acid.

**Figure 9:**
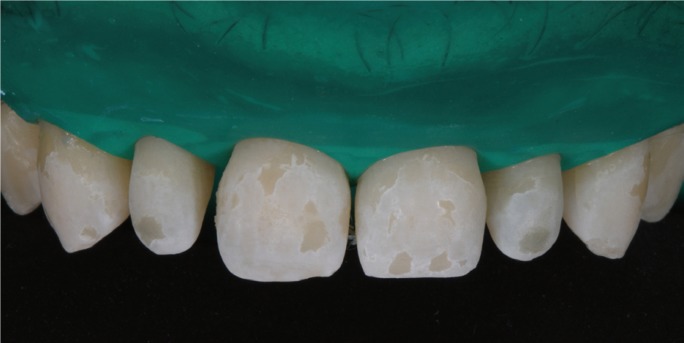
Appearance of teeth after etching, washing and drying.

**Figure 10:**
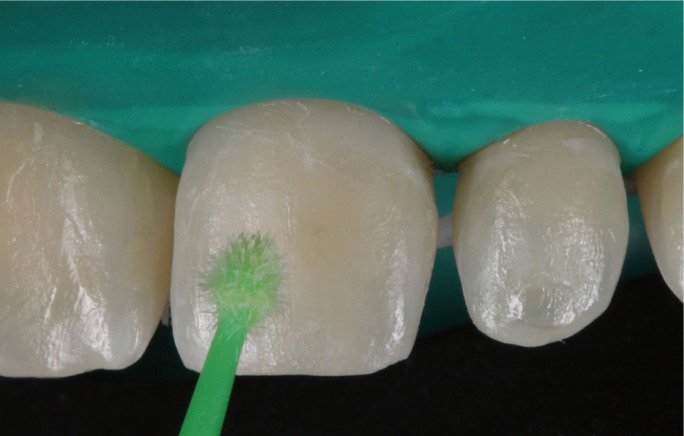
Applying adhesive to teeth.

**Figure 11:**
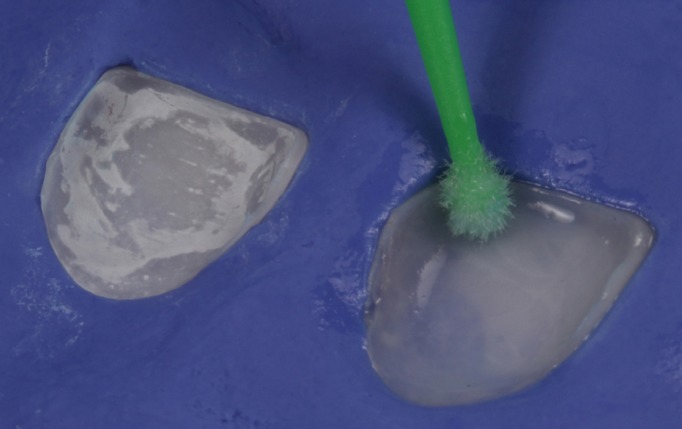
Applying silane to veneers.

**Figure 12:**
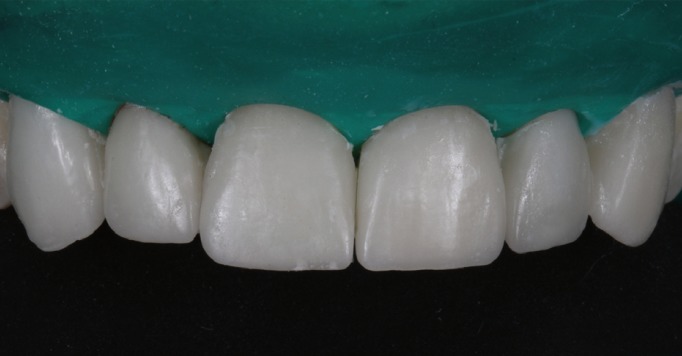
View of cemented veneers before removal of excess cement.

At the second visit, the fit of the veneers in the mouth was checked using glycerin gel (LiquidStrip®, Ivoclar Vivadent, Schaan, Lichtenstein) (Fig. 5), as well as the esthetic effect and gingival displacements (symmetrical scalloping). After checking that dental fit and gingival adaptation were correct, the color shade of the cement was selected using Try-in pastes (Variolink Esthetic, Ivoclar Vivadent) (2,3). Firstly, the teeth were isolated with a rubber dam with a mean thickness of 0.18mm (4) (Dentaflux, Ma-drid, Spain) (Fig. 6), and the teeth were etched with 37% orthophosphoric acid etching gel (Total Etch, Ivoclar Vivadent) for 30 seconds (Fig. 7) and the veneers with 5% hydrofluoric (IPS Ceramic Etching Gel, Ivoclar Vivadent) for 1 minute (Fig. 8). Both the teeth and the veneers were then washed and dried (Fig. 9). Adhesive was applied to the dental enamel (Adhese Universal, Ivoclar Vivadent) and polymerized for 10 seconds (Bluephase G2 curing light, Ivoclar Vivadent) (Fig. 10), and a silane coupling agent (Monobond plus, Ivoclar Vivadent) was applied to the internal face of each veneer, followed by the adhesive (Adhese Universal, Ivoclar Vivadent) without undergoing polymerization (Fig. 11) (4). Then, the veneers were cemented with photpolymerizable resin cement (Variolink Esthetic LC Light, Ivoclar Vivadent) one by one, starting with the central teeth, fitting each veneer in place and polymerizing for 2 seconds in order to remove excess cement. Afterwards full polymerization was performed for 60 seconds per tooth (Fig. 12). Lastly, any remaining excess cement was removed with a scalpel blade (4).

**Figure 13:**
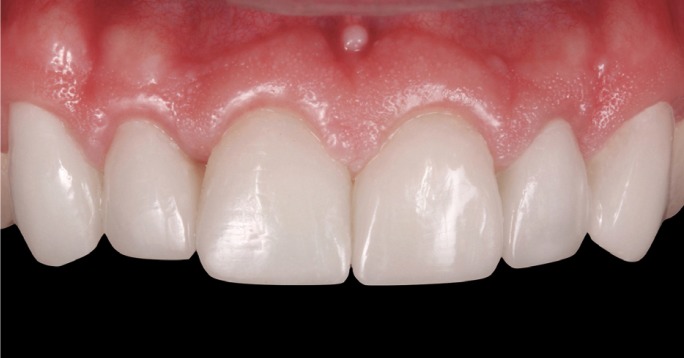
View of veneers at 6-month follow-up visit.

The patient was recalled to the clinic after 15 days, 3 and 6 months to assess the health of the soft tissues, checking that gingival scalloping adjacent to veneers was symmetrical and that prosthetic esthetics were optimal (Fig. 13).

## Discussion

At the outset of the present case, we observed that the asymmetry of the patient’s gingival margins could be regularized by means of several different treatment options. In a case such as this, the classic option consists of performing gingivectomy or crown enlargement followed by prosthetic rehabilitation (3). However, after periodontal and radiographic examination, we observed adequate bone levels and it was clear that major modifications to soft tissues were unnecessary. We opted to apply BOPT philosophy, which would make it possible to modify the height of the gingival margin without any need for surgery, simply by modifying the emergence profile to make it more concave or more convex, which would allow the gums to thicken and adapt to the new shapes. In this way, it was possible to achieve greater gingival stability in the medium and long terms, improve the restorations’ emergence profiles, facilitate oral hygiene maintenance, and create a more natural appearance.

Furthermore, this approach offers several additional advantages: 1. It allows controlled invasion of the gingival groove; 2. The possibility of positioning the prosthetic termination line at different levels inside the groove without affecting marginal adaptation; 3. An optimal restoration-tooth relationship; 4. It is possible to correct the cementoenamel junction (CEJ) on non-prepared teeth (or eliminate the finish line on prepared teeth) (4,5).

The patient presented microdontia and diastemata and the case could be treated without any kind of dental preparation as it met all the requirements for applying veneers without any tooth reduction by milling (2,5). In this way, it was possible to conserve more of the dental structure and treatment was less invasive and pain-free. Enamel conservation is important, due to the fact that adhesion to enamel is better and creates structural unity, whereby the tooth and restoration act as a unit, optimizing strength and long-term durability. Furthermore, many patients are reluctant to allow the elimination of healthy dental material (1,6-8). With no tooth preparation, the shapes of the teeth remain unaltered avoiding the need for provisional restorations and problems of dentin sensitivity. So this type of treatment meets patient demands for maximum conservation (9-13).

As for the choice of materials, it was decided to fabricate the veneers from a hybrid ceramic. These materials combine the versatility of resins – increased elasticity and less risk of fracture during cementation – with the durability and esthetics of ceramics (14,15). They are less fragile and enjoy an excellent milling capacity and their edges are stable. In addition, they would appear to be structurally more reliable than manually processed restorations (16).

VITA Enamic HT was used in the present case, a material intended for CAD/CAM processing, formed of a ceramic matrix (86%) infiltrated with a resin polymer (14%). Due to its dual properties, this material presents a high load capacity after adhesion to dental enamel, absorbing intraoral forces very well. The polymer content prevents against cracking. Thanks to its high load capacity and elasticity, it was possible to make restorations of a very reduced thickness. The material has abrasion behavior that is very similar to dental enamel so that it does not damage the antagonist teeth and its elastic modulus is similar to dentin. Its high translucency allows good light conductivity and so perfect visual integration.

## Conclusions

The use of ultra-fine micro-veneers makes it possible to treat cases presenting diastemata in the anterior region.

Applying BOPT principles allows gingival modeling without the need for any type of pre-prosthetic surgery.

Hybrid ceramics would appear to be an excellent alternative to feldspathic ceramics in this type of case, although longitudinal medium-long term studies are needed to confirm the correct behavior of this material in the oral medium over time.
